# Progress in Skin Diseases

**Published:** 1902-10-04

**Authors:** 


### Progress in Skin Diseases.

(*Concluded from page* 452.)

**Leprosy.**—D. Thomson[8] reports on the compulsory detention hospital in New South Wales, where 69 patients have been treated and 11 remain as inmates. In seven cases intra-muscular injections of mercury perchloride were tried but given up either on account of stomatitis or of the refusal of patients to go on with them. However, some improvement was noticed while the treatment was employed, and further trials are therefore to be desired, and some modification of it may prove to be of real value. T. J. Tonkin[9] found the disease very prevalent in the Soudan and Northern Nigeria. The infection seems to be conveyed by clothing, which is never washed, but transferred from one owner to another, even if stiff with leprous exudation. The disease generally commences on some bony prominence which is chafed by the clothes. He regards the deficiency of nitrogenous food as the chief predisposing cause of the disease both in this and in other leprous districts. He finds no evidence in favour of heredity as playing any part in causing it. Jonathan Hutchinson, in speaking of South Africa, lays down that the disease was almost or entirely unknown before the Dutch settlement and foundation of a salt fish factory at Capetown. Salt fish and rice became the staple food of the farmers and their slaves, and the disease has since spread over the whole of what is now British South Africa. The disease rarely occurs in the towns but is found in the country among the fish-eating labourers. In Natal it seems to spread among the Kaffirs by contagion, especially by eating from the hands of leprous persons. Thus he found infected Kaffir lads who had never left their homes or eaten salt fish, but lived with leprous elder persons in healthy situations, and some of these latter could always be shown to have stayed in a fish eating district. The bacillus, he thinks, always enters by the stomach and never through an abrasion of the skin. Thus there is nothing of the nature of a primary sore, and the first lesions are bilateral, showing a blood infection. This infection thus enters through the stomach and not by contagion pure and simple, and occurs only among uncleanly feeders. It rarely spreads between husband and wife, or among cleanly people. In the discussion of these papers[10] it was pointed out that in spite of salt fish there were only a seventh part of the lepers in Norway which could be counted 50 years ago, and that in spite of heredity the Norwegians in America develop no leprosy. G. Thin mentioned that the bacillus had been cultivated outside the body, but it was necessary to take it at a time of fever. Mauson referred to its cultivation on a medium formed from salt fish by some of the prisoners in Ceylon, and denied that the first lesions are symmetrical. Heron mentioned that 162 lepers in India had never eaten fish, and Tonkin spoke of the rareness of fish in the Soudan. Against the theory of simple contagion Hutchinson showed that the failure of attempt at inoculation was a strong argument. In Cashmir a large proportion of the lepers, says E. F. Neve,[11] deny that they have eaten fish. The disease is rare in the city of Srinagar, and among Hindus, women, and the boatmen, but it abounds among the herdsmen in mountainous districts, which again does not favour the fish theory. Children at birth are free from the disease but if brought up with their parents they become affected. It is possible, he thinks, that fish, cheese, milk, and other articles of food may be sources of danger, and he recommends segregation and sanitary precautions, much the same as would be used against tuberculosis, with which the disease has many points of resemblance.

**Recurrent Herpes.**—A patient who has had one attack of herpes is generally free for the rest of his life, but there are rare instances in which the disease recurs year after year. How these differ in pathology is not clear. They certainly have all the symptoms of true herpes zoster[12]. Grindon has collected 61 cases. Malignon describes an attack of the ophthalmic nerve which recurred three or four times a year from childhood. G. Pernet records a case where four attacks in the sub-maxillary and inter-costal regions were noted, and Beatty mentions five attacks in one patient, the parts affected being the toes and genital regions. Hirtz and Salomon recently reported one where nine outbreaks of vesicles on the buttocks occurred with much pain. Though chiefly affecting the right side, there seem to have been a few vesicles on the left also. Pain of a neuralgic type preceded the attacks.

**Staphylococcus Vaccine.** — A most interesting paper by Professor A. E. Wright[13] opens up a wide field for the employment of vaccines. He details the results of staphylococcal vaccines in sycosis boils and acne of long standing and most intractable character. Some marked successes were obtained, and the deductions he draws are even more important with a view to the possibilities of antitoxic treatment. For some reason anti-staphylococcal serum has not been put into the market, but in his cases Wright employed a vaccine made by sterilising a culture at 65° for 20 minutes. He calls attention to the fact that after the use of such a vaccine in moderate doses there is a stage of diminished bacteridal power or diminished resistance for a few days, followed by a positive stage of increased power. We are indeed taking temporarily away the patient's power of resistance that he may receive it again with usury. His first patient had suffered for seven years from crops of boils with eczema and sycosis in spite of various treatment. Staphylococcus albus was found in pure culture in the boils. Three inoculations were made. A complete cure followed, and his face has now been for 12 months quite clean and free from eruption. The ratio of the number of cocci destroyed by each of the patient's polynuclear blood-cells to that in normal individuals (Leishman's measure of phagocytosis) has run as high as 1·45 : 1. In a second case of furunculosis good results were at last obtained, but the ratio could not be raised higher than ·75 : 1. In a third case where persistent boils were cured the phagocytic ratio was raised from ·38 : 1 to even 1·2 : ·1 and higher, the clinical improvement closely agreeing with the rise of the ratio. Wright suggests that the method may be of use in surgical operations where asepsis is impossible as a means of raising the patient's resisting power beforehand. Again, in old ulcers, sinuses, septic vaccination and other wounds, it may be of great service. Unlike tuberculin vaccination, when the nidus is broken up by the vaccine reaction the staphylococci are killed.

[8] Brit. Med. Jour., April 26. [9] Lancet, May 31. [10] Lancet, June 14. [11] Brit. Med. Jour., May 3. [12] Lancet, April 12. [13] Lancet, March 29.

